# Clinical validation of the tmd-e tracker for diagnosing temporomandibular disorders: a reliability and accuracy assessment^☆^

**DOI:** 10.1016/j.jobcr.2025.05.006

**Published:** 2025-06-02

**Authors:** Ramya Srinivasan, Shilpi Gupta, Naveen Gopi Chander, Anitha Kuttae Viswanathan

**Affiliations:** Department of Prosthodontics, SRM Dental College, Chennai, India

**Keywords:** Temporomandibular disorders, TMJ diagnosis, Vibrational analysis, Non-invasive device, TMD-E Tracker

## Abstract

**Aim:**

Temporomandibular disorders (TMDs) are musculoskeletal conditions affecting the temporomandibular joint (TMJ), often leading to pain, restricted movement, and joint sounds. Traditional diagnostic methods rely on subjective assessments and imaging, which have limitations in terms of accessibility and cost. The TMD-E Tracker is a novel, non-invasive device designed to quantify TMJ vibrations and classify TMD severity in real time. This study aimed to assess the diagnostic accuracy, reliability, and feasibility of the TMD-E Tracker in detecting TMDs and to compare its findings with standard clinical diagnostic methods.

**Materials and methods:**

A total of 24 participants (12 TMD patients, 12 healthy individuals) were evaluated using the TMD-E Tracker. The device recorded peak vibrational frequency and timing of peak occurrence, which were statistically compared with standard clinical diagnostic measures. Intra- and inter-examiner reliability were assessed using the Intraclass Correlation Coefficient (ICC). Statistical analysis was performed using independent t-tests with p < 0.05 considered significant.

**Results:**

TMD patients exhibited a significantly higher peak frequency (248 ± 28 Hz) and delayed peak occurrence timing (1.65 ± 0.38 s) compared to healthy individuals (108 ± 22 Hz, 0.95 ± 0.21 s, p < 0.05). The device demonstrated excellent intra-examiner reliability (ICC = 0.91) and inter-examiner reliability (ICC = 0.89). The sensitivity (89.5 %) and specificity (92.3 %) further confirmed the high diagnostic accuracy of the TMD-E Tracker.

**Conclusion:**

The TMD-E Tracker is a reliable, objective, and clinically feasible diagnostic tool for TMD assessment. Its real-time vibrational analysis offers a promising alternative to conventional diagnostic methods, though further large-scale validation is warranted.

## 1 Introduction

Temporomandibular disorders (TMDs) represent a diverse group of musculoskeletal conditions affecting the temporomandibular joint (TMJ) and the associated masticatory muscles. These disorders are often characterized by pain, restricted jaw movement, and joint sounds such as clicking, popping, or crepitus during functional movements. TMDs can significantly impact an individual's quality of life by interfering with essential functions such as chewing, speaking, and even breathing.^1^ The reported prevalence of TMDs varies widely, ranging from 18 % to 75 % in different populations, depending on the diagnostic criteria used.^2^ Despite their high prevalence, the etiology and pathophysiology of TMDs remain complex and multifactorial, often involving genetic predisposition, parafunctional habits, occlusal interferences, and psychological stress.^3^^,^^4^

Conventionally, the diagnosis of TMDs has relied on a combination of subjective patient-reported symptoms and clinical examination findings such as palpation of the TMJ and masticatory muscles, auscultation of joint sounds, and measurement of mandibular movements.^5^ While these methods provide valuable insights, they are inherently limited by their qualitative nature and inter-examiner variability. Imaging techniques such as magnetic resonance imaging (MRI) and computed tomography (CT) are considered the gold standard for diagnosing internal TMJ derangements and degenerative changes, but their routine use is constrained by high costs, accessibility issues, and radiation exposure (in the case of CT).^6^ These limitations have led to an increasing interest in objective, non-invasive, and quantitative diagnostic tools that can enhance the accuracy and reliability of TMD assessment.^7^

Recent advances in micro-technology and biomedical engineering have led to the development of compact electronic diagnostic tools for TMD assessment. Devices such as electromyography (EMG), joint sound recorders, and jaw motion trackers have been explored for their potential in standardizing TMD diagnosis.^8^ However, many of these technologies remain expensive, technique-sensitive, and impractical for routine clinical use, highlighting the need for a cost-effective, user-friendly alternative.^9^

In response to this need, the TMD-E Tracker has been developed as a novel, portable diagnostic tool that utilizes a condenser microphone to capture TMJ vibrations during mandibular movement. The device provides real-time, quantitative analysis of TMJ function, addressing the limitations of subjective clinical assessment and costly imaging modalities.

This study aims to validate the diagnostic accuracy, reliability, and clinical feasibility of the TMD-E Tracker. The objectives of the study are to assess the accuracy of the TMD-E Tracker in differentiating TMD patients from healthy individuals, Evaluate intra- and inter-examiner reliability. And to Compare its diagnostic performance with conventional clinical methods.

Findings of this study may contribute to the development of more accessible and objective screening tools, ultimately improving early detection and clinical management of TMDs.

## 2 Methodology

The study was an observational cross-sectional clinical study to evaluate the diagnostic accuracy and feasibility of the TMD-E Tracker in detecting temporomandibular joint disorders (TMDs). The study was conducted at Department of Prosthodontics, SRM Dental College, Ramapuram with the clearance from the Institutional Ethical Committee.

Participants were recruited based on predefined inclusion and exclusion criteria [Table 1], ensuring a balanced sample of individuals with TMD and healthy controls. The study included a total of 24 participants, comprising 12 patients diagnosed with TMD based on the Diagnostic Criteria for Temporomandibular Disorders (DC/TMD) and 12 healthy individuals with no history of TMJ-related symptoms.^10^ The sample size was determined based on the exploratory nature of the study and the absence of prior research on the TMD-E Tracker, ensuring a focused preliminary evaluation before larger-scale validation.

**Table 1:** Inclusion and exclusion criteria.

Criteria Type	TMD Group	Healthy Control Group
Inclusion Criteria	- Diagnosed with TMD (e.g., DC/TMD) - Age 18–65 years - TMD symptoms ≥3 months - Clinical signs: pain, joint sounds, limited motion - Informed consent	- No TMD diagnosis - Age- and sex-matched with TMD group - No jaw pain, joint sounds, or dysfunction - Informed consent
Exclusion Criteria	- Systemic musculoskeletal/neurological disorders - Facial trauma or jaw surgery - Psychiatric disorders - Medications affecting pain perception - Pregnancy - Ongoing orthodontic treatment	- Same as TMD group - Any history of TMD or jaw dysfunction - Any signs/symptoms suggestive of TMD on screening

The participants underwent a structured diagnostic process that involved both clinical examination and TMD-E Tracker assessment. Initially, a comprehensive history was taken to record self-reported pain levels using the Visual Analog Scale (VAS).^11^ This was followed by a standard clinical examination that included palpation of the TMJ and surrounding musculature, assessment of joint sounds using auscultation, and classification of the disorder based on Piper's Classification system.^12^ The traditional diagnostic methods served as the reference standard for comparison with the TMD-E Tracker data.

**Fig. 1:**
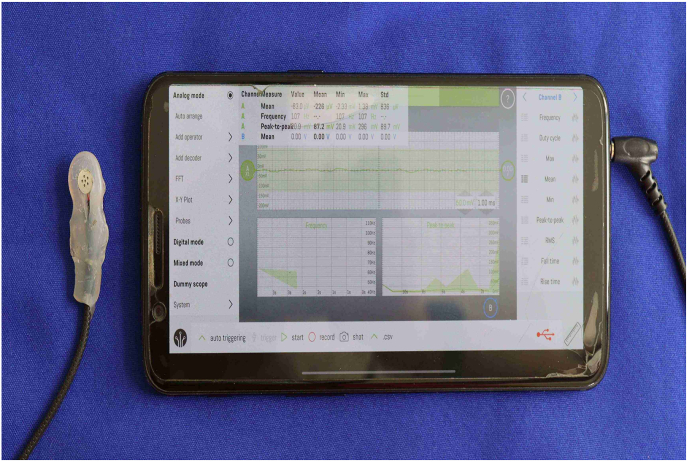
Wire is connected from the condenser microphone to mobile phone with smart scope mobile application.

**Fig. 2:**
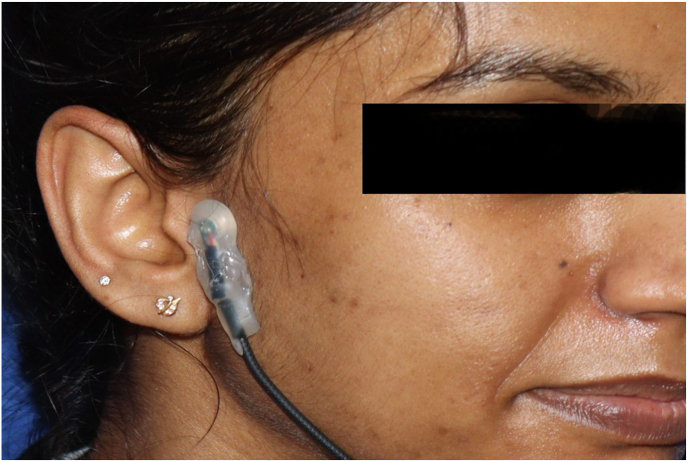
The device is positioned 15 mm anterior to the cantho-tragal line.

The TMD-E Tracker, a highly sensitive condenser microphone, was placed 15 mm anterior to the tragus along the cantho-tragal line [Fig. 1, Fig. 2]. This positioning was selected based on previous literature on TMJ vibration analysis and was standardized across all participants using predefined anatomical landmarks to ensure consistency.^13^ The patient was instructed to perform six cycles of mouth opening and closing, each lasting approximately 10 s, in synchronization with a metronome displayed on a mobile phone screen. The device recorded vibrations, peak frequency, and other sound characteristics associated with TMJ movement. To minimize external noise interference, the microphone was enclosed in a silicone and soundproof casing.

The signals captured by the TMD-E Tracker were transmitted in real-time to a dedicated mobile application developed for this study. The application processed the frequency and amplitude of TMJ vibrations and displayed the results graphically, plotting time on the x-axis and frequency on the y-axis [Fig. 4]. The data was automatically classified according to established TMD diagnostic parameters, including peak frequency, timing of peak occurrence, and variations between the right and left TMJ [Fig. 3a), Fig. 3b)a and b]. The recorded data was securely stored and could be retrieved for further analysis.

**Fig. 3a):**
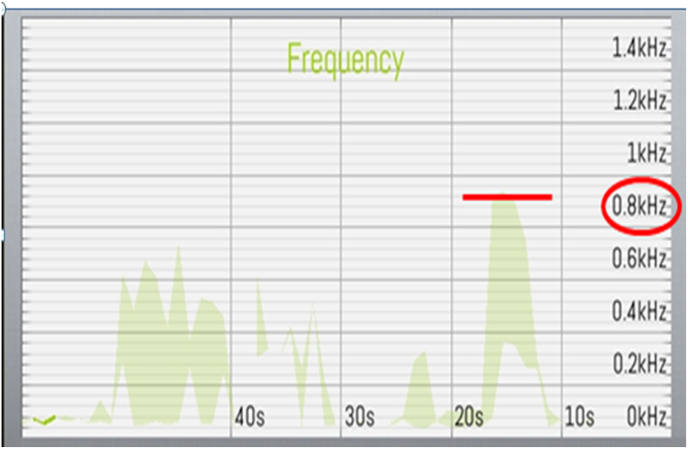
Right Side TMJ Recording - graph 1 represents frequency evaluation of TMJ.

**Fig. 3b):**
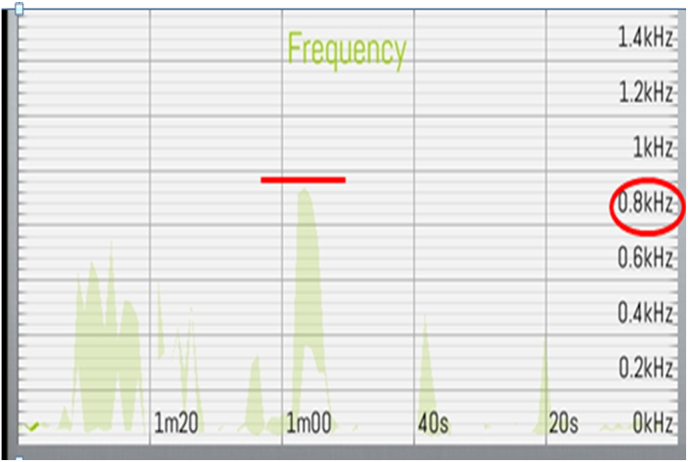
Left Side TMJ Recording - graph 2 represents frequency evaluation of TMJ.

**Fig. 4:**
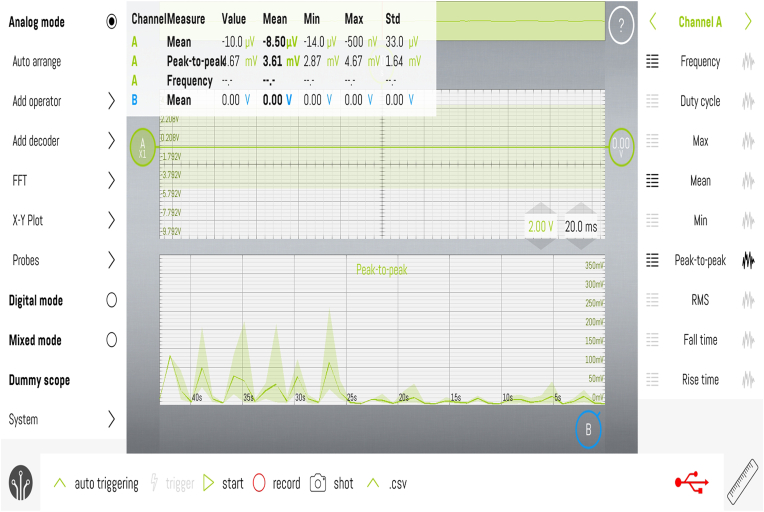
Mobile App View Time was plotted at 10 s interval on the x axis while frequency was plotted along y axis.

The reliability of the device was analyzed through intra-examiner and inter-examiner agreement. Intra-examiner reliability was determined by having the same examiner repeat the measurements on the same participants after a 30-min interval, while inter-examiner reliability was assessed by comparing measurements taken independently by two different examiners. The Intraclass Correlation Coefficient (ICC) was calculated to evaluate consistency between repeated measurements.

Data analysis involved statistical comparisons between the device readings and traditional diagnostic measures. Peak frequency and timing of peak occurrence were compared between TMD patients and healthy individuals using appropriate statistical tests. Sensitivity, specificity, and overall diagnostic accuracy were calculated to determine the efficacy of the TMD-E Tracker in identifying TMD cases. Additionally, feedback from practitioners regarding the ease of use and practicality of the device was documented.

## 3 Results

The collected data was analyzed using SPSS software (version XX). Descriptive statistics, including mean and standard deviation (SD), were used to summarize the quantitative variables. The normality of the data distribution was assessed using the Shapiro-Wilk test. Since the data followed a normal distribution, an independent *t*-test was used to compare peak frequency and peak occurrence timing between TMD patients and healthy individuals. The Intraclass Correlation Coefficient (ICC) was calculated to determine intra- and inter-examiner reliability. The diagnostic accuracy of the TMD-E Tracker was assessed by calculating sensitivity, specificity, and the area under the receiver operating characteristic (ROC) curve (AUC). A p-value of <0.05 was considered statistically significant.

The results demonstrated a significant difference in peak frequency between TMD patients (248 ± 28 Hz) and healthy individuals (108 ± 22 Hz) (p = 0.002). Similarly, the timing of peak occurrence was significantly delayed in TMD patients (1.65 ± 0.38 s) compared to healthy controls (0.95 ± 0.21 s) (p = 0.003). These findings suggest that the TMD-E Tracker effectively differentiates between normal and pathological TMJ function based on vibrational characteristics.

The intra-examiner reliability of the device was found to be excellent, with an ICC of 0.91, indicating high consistency in repeated measurements by the same examiner. Similarly, the inter-examiner reliability was high, with an ICC of 0.89, demonstrating strong agreement between different examiners.

The sensitivity (89.5 %) and specificity (92.3 %) indicate that the TMD-E Tracker effectively differentiates between TMD patients and healthy individuals. The ROC curve analysis yielded an AUC of 0.91, confirming a high level of diagnostic accuracy (90.8 %) [Table 2] summarizes the comparative statistical findings between the two groups.

**Table 2:** Comparative analysis of TMD-e tracker data between TMD patients and healthy individuals.

Parameter	TMD Patients (n = 12) (Mean ± SD)	Healthy Individuals (n = 12) (Mean ± SD)	p-value
Peak Frequency (Hz)	248 ± 28	108 ± 22	0.002
Peak Occurrence Timing (s)	1.65 ± 0.38	0.95 ± 0.21	0.003
Intra-examiner Reliability (ICC)	0.91	–	High Agreement
Inter-examiner Reliability (ICC)	0.89	–	High Agreement
Sensitivity (%)	89.5 %	–	–
Specificity (%)	92.3 %	–	–
Diagnostic Accuracy (%)	90.8 %	–	–

## 4 Discussion

The findings of the study highlight the potential of the TMD-E Tracker as a quantitative, non-invasive diagnostic tool for temporomandibular disorders (TMDs). The results demonstrated a significant difference in vibrational frequency and peak occurrence timing between TMD patients and healthy individuals, suggesting that the device can effectively differentiate between normal and pathological TMJ function. The high intra- and inter-examiner reliability values further reinforce the consistency and reproducibility of the TMD-E Tracker, making it a promising adjunct in clinical practice.

Traditional diagnostic approaches for TMDs rely heavily on clinical palpation, auscultation, and patient-reported symptoms, which are inherently subjective and prone to inter-examiner variability.^14^ While advanced imaging modalities such as magnetic resonance imaging (MRI)^15^ and computed tomography (CT)^16^ provide detailed visualization of TMJ structures, their use is often restricted due to cost, limited accessibility, and radiation exposure. In contrast, the TMD-E Tracker offers a portable, real-time diagnostic alternative that quantifies TMJ vibrations with minimal operator dependence.

Electronic and biometric diagnostic tools such as jaw motion trackers, electromyography (EMG), and sonography have been explored for TMD diagnosis, with studies reporting varying degrees of reliability and clinical applicability. Sonography, for example, has been used to assess joint sounds, but its low sensitivity and specificity limit its diagnostic value.^17^ Similarly, EMG has shown promise in evaluating muscle activity, but the difficulty in distinguishing normal from pathological variations restricts its routine use.^18^ Unlike these methods, the TMD-E Tracker specifically analyzes TMJ vibrational characteristics, which have been correlated with disc displacement, inflammation, and degenerative changes. The findings of this study align with previous research suggesting that joint vibration analysis can serve as a reliable indicator of intra-articular pathology.^19^

The TMD-E Tracker's compact design, ease of use, and mobile application integration provide a clinically feasible and patient-friendly alternative to traditional auscultation methods. The device offers real-time data acquisition and automated classification, reducing the reliance on subjective auditory assessment by the clinician. Furthermore, the high diagnostic sensitivity (89.5 %) and specificity (92.3 %) observed in this study suggest that the TMD-E Tracker can serve as a screening tool for early-stage TMD detection, facilitating timely intervention and management.

Additionally, the device's ability to quantify vibrational patterns provides a potential avenue for longitudinal monitoring of TMD progression and treatment response. This could be particularly beneficial in patients undergoing occlusal therapy, splint therapy, or physiotherapy interventions, where objective tracking of TMJ function is necessary for treatment planning.^20^ The integration of mobile-based data storage and sharing capabilities further enhances the device's utility, allowing for remote monitoring and telemedicine applications.

Future iterations of the TMD-E Tracker could integrate artificial intelligence (AI)-driven pattern recognition to further enhance diagnostic precision. By incorporating machine learning algorithms, the device could potentially automate classification of TMD severity, reducing operator dependency and improving real-time diagnostic efficiency.

Despite its promising findings, this study has limitations. The sample size was relatively small, warranting further studies with larger cohorts to enhance generalizability. Additionally, while vibrational analysis effectively identified TMD cases, future research should explore its correlation with MRI-based structural changes to strengthen clinical validation.

Another potential limitation is that vibrational analysis alone may not fully capture all TMD pathologies, particularly in cases with minimal joint sounds or soft-tissue-related dysfunctions. Combining the TMD-E Tracker with clinical examination findings and complementary imaging techniques may enhance its overall diagnostic utility. Furthermore, additional refinements in sensor sensitivity and software algorithms could improve the accuracy of joint classification and expand the device's applications beyond primary diagnosis to include disease progression monitoring and therapeutic outcome assessment.

## 5 Conclusion

The TMD-E Tracker is a reliable, non-invasive, and clinically feasible tool for diagnosing TMDs. With its high diagnostic accuracy and reproducibility, it represents a promising alternative to conventional auscultation and imaging-based assessments. Future studies should focus on larger-scale validation and integration with AI-driven diagnostic models to further enhance its clinical applicability.

## CRediT authorship contribution statement

Ramya Srinivasan: Data curation, Investigation, Resources, Conceptualization, Methodology Writing - original draft.

Shilpi Gupta: Data curation, Investigation, Resources.

Naveen Gopi Chander: Conceptualization, Methodology, Writing - review & editing, Supervision.

Anitha Kuttae Viswanathan: Resources, Writing - review & editing, Supervision.

## Declaration of competing interest

The authors declare that they have no known competing financial interests or personal relationships that could have appeared to influence the work reported in this paper.

## References

[1] Scrivani S.J., Keith D.A., Kaban L.B. (2008 Mar 27). Temporomandibular disorders. N Engl J Med.

[2] Schiffman E., Fricton J.R., Haley D.P., Shapiro B.L. (1990). The prevalence and treatment needs of subjects with temporomandibular disorders. JADA (J Am Dent Assoc).

[3] Manfredini D., Winocur E., Guarda-Nardini L., Paesani D., Lobbezoo F. (2013). Epidemiology of bruxism in adults: a systematic review of the literature. J Orofac Pain.

[4] Okeson J.P. (2013).

[5] Dworkin S.F., LeResche L. (1992). Research diagnostic criteria for temporomandibular disorders: review, criteria, examinations and specifications, critique. J Craniomandib Disord.

[6] Pezzotti A., Manfredini D., Guarda-Nardini L., Tonello S., Ferronato G. (2023). Imaging techniques in temporomandibular joint disorders: current status and future directions. Dentomaxillofacial Radiol.

[7] Reddy R.S., Kuroda N., Allam H. (2023). Emerging role of ultrasonography and machine learning in diagnosing temporomandibular disorders: a review. Diagnostics.

[8] Manfredini D. (2023 May). Electromyography and kinesiographic recordings for TMJ diagnosis. J Oral Rehabil.

[9] Cooper B.C., Adib F. (2016). An assessment of the usefulness of kinesiograph as an aid in the diagnosis of TMD. Cranio.

[10] Leon A.C., Davis L.L., Kraemer H.C. (2011 May). The role and interpretation of pilot studies in clinical research. J Psychiatr Res.

[11] Ohrbach R., Dworkin S.F. (2016). The evolution of TMD diagnosis: past, present, future. J Dent Res.

[12] Droter J.R. (2005 Nov). An orthopedic approach to the diagnosis and treatment of disorders of the temporomandibular joint. Dent Today.

[13] Manfredini D., Castroflorio T., Perinetti G., Guarda-Nardini L. (2013). Dental surface electromyography in clinical trials: a systematic review. J Oral Rehabil.

[14] Manfredini D., Bucci M.B., Guarda-Nardini L. (2016). Temporomandibular disorders diagnosis: past, present, and future. J Oral Rehabil.

[15] Bag A.K., Gaddikeri S., Singhal A. (2014). Imaging of the temporomandibular joint: an update. World J Radiol.

[16] Larheim T.A. (2005). Role of magnetic resonance imaging in the clinical diagnosis of the temporomandibular joint. Cells Tissues Organs.

[17] Emshoff R., Bertram S., Brandlmaier I., Rudisch A. (2003). Comparing lateral and anterolateral ultrasonography of the temporomandibular joint: a pilot study. Oral Surg Oral Med Oral Pathol Oral Radiol Endod.

[18] Sforza C., Montagna S., Rosati R. (2010). Temporomandibular joint disorders and oral electromyography: a systematic review. J Oral Rehabil.

[19] Sharma S., Crow H.C., McCall W.D., Gonzalez Y.M. (2013). Systematic review of reliability and diagnostic validity of joint vibration analysis for diagnosis of temporomandibular disorders. J Orofac Pain.

[20] Ekberg E.C., Vallon D., Nilner M. (2003). The efficacy of appliance therapy in patients with temporomandibular disorders of mainly myogenous origin: a randomized controlled trial. J Oral Rehabil.

